# Supplementary material for: Zeb1-Hdac2-eNOS circuitry identifies early cardiovascular precursors in naive mouse embryonic stem cells
Source: Nat Commun. 2018 Mar 29;9:1281. doi: 10.1038/s41467-018-03668-0 (PMC5876398; doi:10.1038/s41467-018-03668-0)
Supplement: Supplementary file 1 — Supplementary Information(PDF 2564 kb) [file 41467_2018_3668_MOESM1_ESM.pdf]

A Zeb1-Hdac2-eNOS circuitry identifies early cardiovascular precursors in naïve mouse embryonic stem cells

Cencioni et al.

Supplementary figures

Supplementary Fig. 1

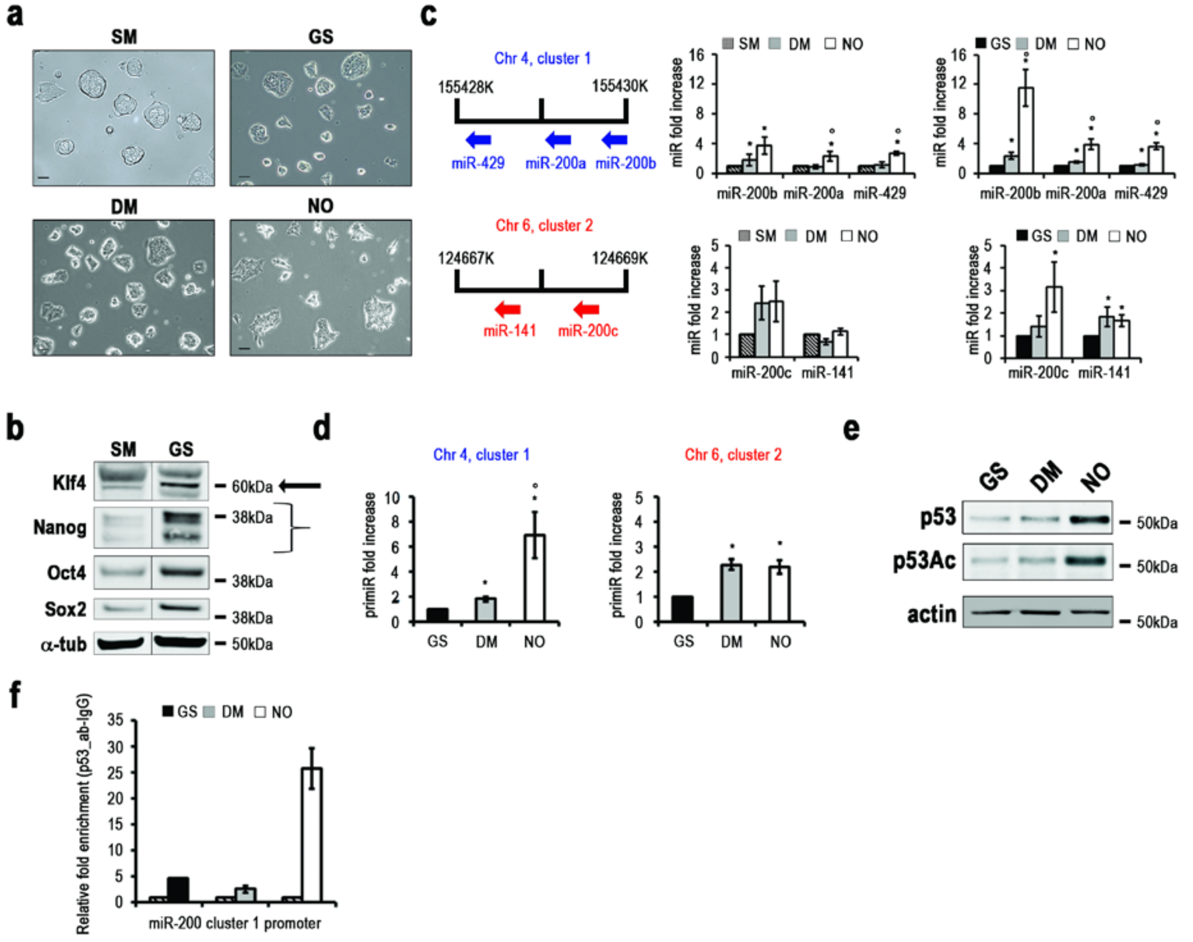

Supplementary Fig. 1 <u>Naïve mESC characterization and response to an exogenous source of NO.</u> (**a**) Representative phase contrast microscopy images of mESC cultured 24 h in SM, GS, DM and NO. Scale bar 10µm. (**b**) Representative western blot analysis of three independent cell extract preparations from mESC cultured 24 h in SM or GS probed with Klf4, Nanog, Oct4 and Sox2 specific antibodies. In each condition, α-tubulin was used as loading control. Full-length blot provided in Supplementary Fig. 12a. (**c**) Left upper and lower panels: schematic representation of miR-200 family genomic organization (cluster 1, blue upper; cluster 2, red lower). Right panel: qRT-PCR analysis of miR-200 family member levels in DM (gray bars) and NO (white bars) expressed as fold-induction versus SM (striped bars) or GS (black bars) respectively (n=8 each time point; *p < 0.05 Vs SM or GS; ° p < 0.05 vs. DM). (**d**) qRT-PCR analysis of miR-200 primiRs from cluster 1 (left panel) or cluster 2 (right panel) in mESC cultured 24h in GS (black bars), DM (grey bars) and NO (white bars). Data are shown as the mean fold increase ±s.e.m. against GS after subtraction of the housekeeping gene p0 signal (n=5; *p < 0.05 vs. GS; °p < 0.05 vs. DM). (**e**) Representative western blot analysis of cell extracts obtained from mESC cultured 24h in GS, DM and NO (n=3 each group) probed with total (p53) or acetylated p53 (p53Ac) antibodies. Actin antibody was used as loading control for each extract. Full-length blot provided in Supplementary Fig. 12b. (**f**) ChIP/qRT-PCR analysis of p53 chromatin binding on miR-200 cluster 1 promoter. Chromatins were extracted from mESC culture 24h in GS (black bar), DM (gray bar) and NO (white bar). Data are shown as mean fold increase ±s.e.m. compared to IgG value (striped bars) after Input normalization (n=3). Data analysed by Kolmogorov-Smirnov test.

Supplementary Fig. 2

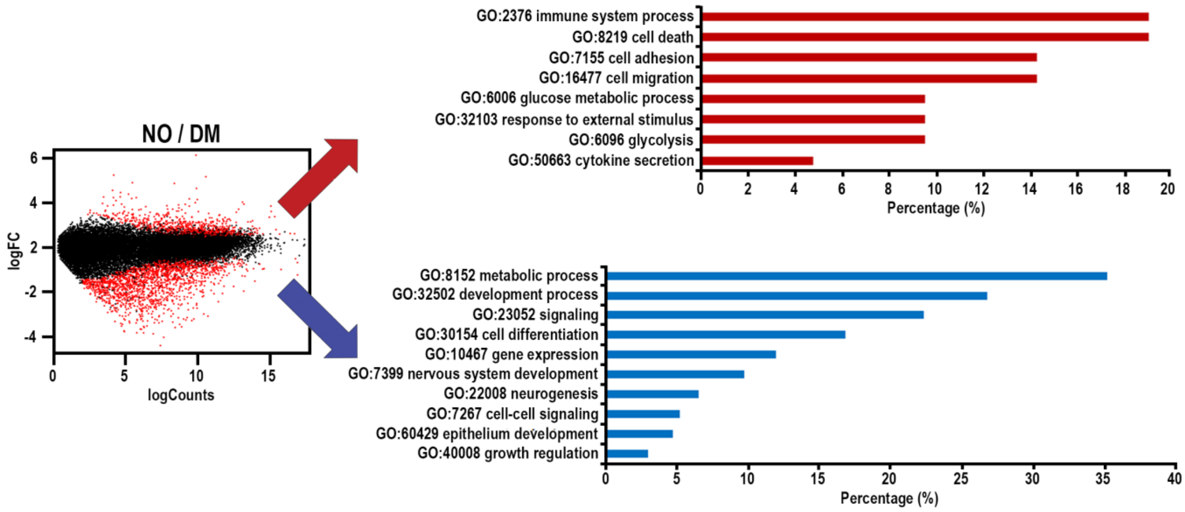

Supplementary Fig. 2 NO-responsive transcripts compared to DM. Left panel: MA plot of differentially regulated genes (DEGs) expressed in mESC cultured for 24h in NO compared to DM condition. Red dots show genes with a padj < 0.05. Right panels: Gene ontology analysis of DEG between mESC cultured in NO and DM. Up-regulated genes depicted in the upper panel, red bar graph; down-regulated genes in the lower panel, blue bar graph.

Supplementary Fig. 3

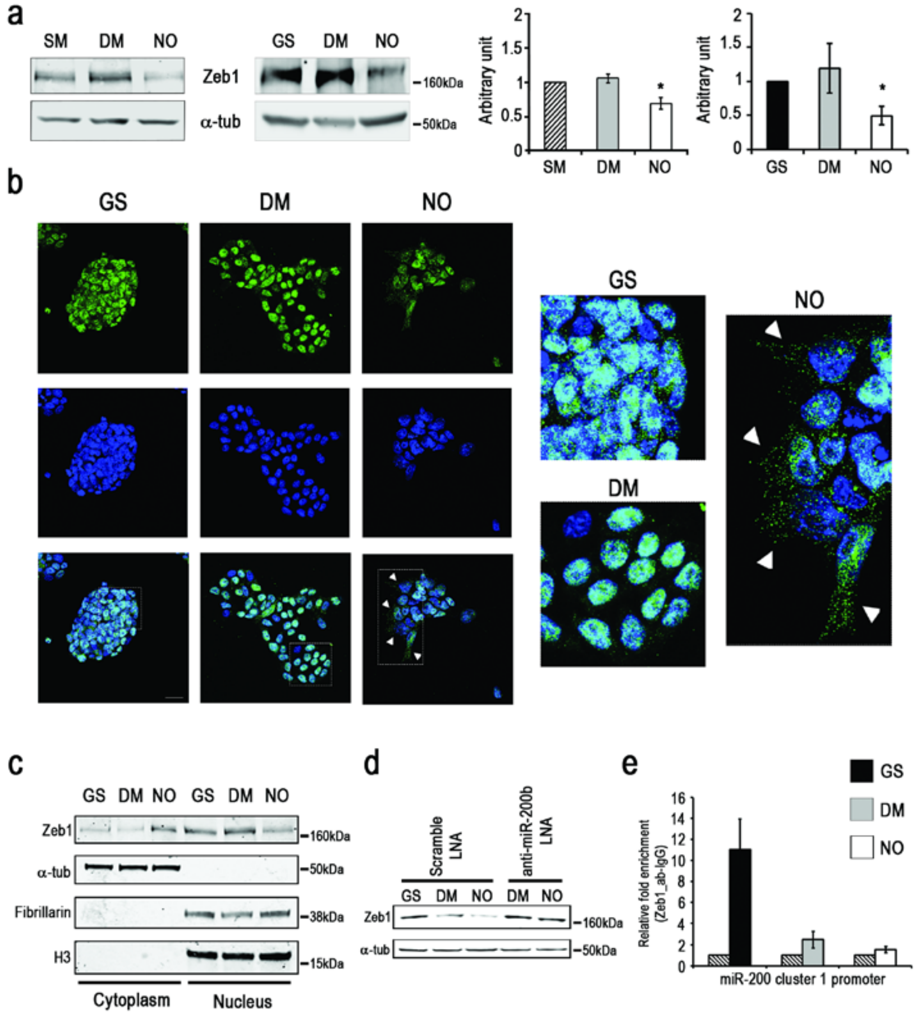

Supplementary Fig. 3 Zeb1 regulation in naïve and differentiating mESC. (**a**) Representative western blotting analysis of cell extracts obtained from mESC cultured 24h in SM, DM and NO (left panel) and GS, DM and NO (right panel) probed with Zeb1 antibody. For each extract, α-tubulin was used as loading control. The experiment was repeated three times. Right panels show the result of densitometric analysis (*$p < 0.05$ vs. stemness condition). Full-length blot provided in Supplementary Fig. 13a. (**b**) Representative confocal microscopy images depicting mESC cultured 24h in GS, DM or NO. Cells were probed by an anti-Zeb1 antibody (green; upper panels). Nuclei were counterstained with TROPRO 3 (blue; middle panels). Merged fluorescence images are shown in the lower panels. On the right, the insets contain enlargements of the selected areas (dashed squares). Scale bar 20µm. (**c**) Representative western blotting analysis of cytoplasm/nuclear fractionation extracts obtained from mESC cultured 24h in GS, DM or NO probed with Zeb1. For cytoplasmic extracts, α-tubulin was used as a loading control, whereas for nuclear extracts Fibrillarin and H3 were used as loading control. The experiment was repeated three times. Full-length blot provided in Supplementary Fig. 13b. (**d**) Representative western blotting analysis of cell extracts obtained from mESC cultured 24h in GM, DM and NO after nucleofection either with Scramble_LNA or anti-miR-200b_LNA probed with Zeb1 antibody. For each extract, α-tubulin was used as loading control. The experiment was repeated three times. Full-length blot provided in Supplementary Fig. 13c. (**e**) ChIP/qRT-PCR analysis of Zeb1 chromatin association to the miR-200 cluster 1 promoter region. Chromatins were extracted from mESC cultured 24h in GS (black bar), DM (gray bar) and NO (white bar). Data are shown as mean fold increase ±s.e.m. compared to IgG value (striped bars) after Input normalization (n=3). Data analysed by Kolmogorov-Smirnov test.

Supplementary Fig. 4

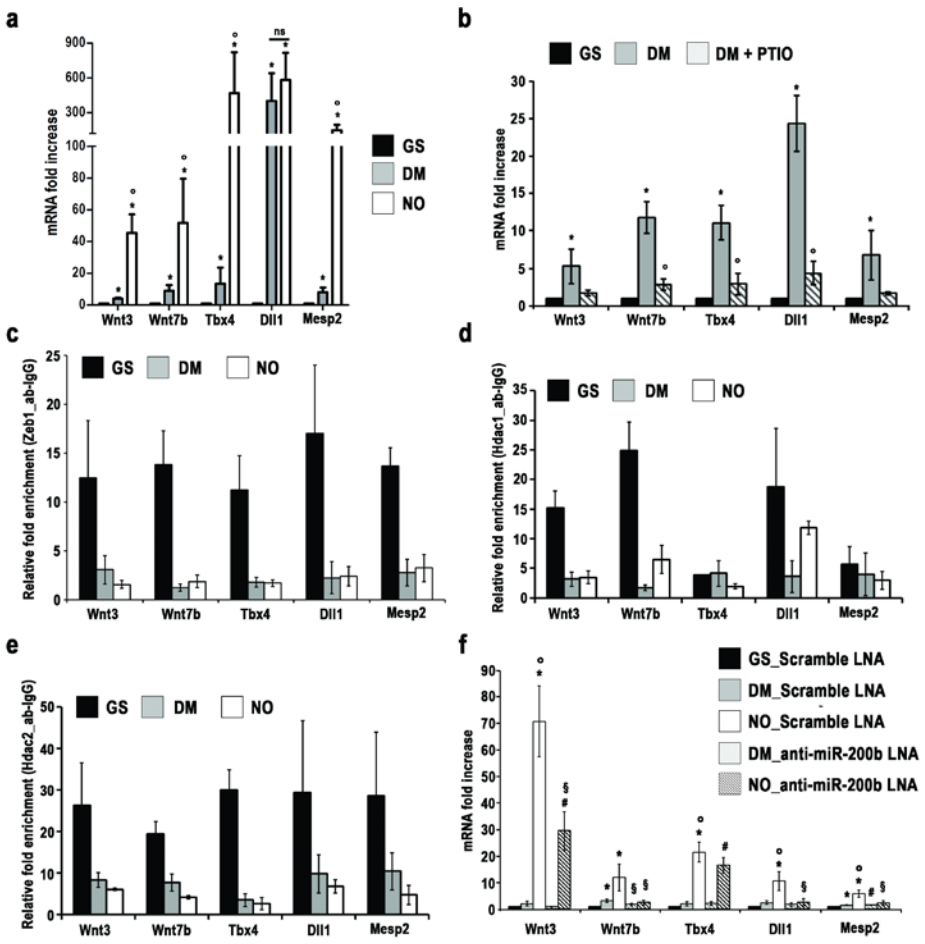

Supplementary Fig. 4 Gene qRT-PCR validation after integration of RNA-seq and ChIP-seq and analysis of miR-200b inhibition. (**a**) mRNA expression analysis of Wnt3, Wnt7b, Tbx4, Dll1 and Mesp2 in GS (black bars), DM (grey bars) or NO (white bars). Data represented as mean fold increase ±s.e.m. compared to GS after subtraction of the housekeeping gene p0 signal (n=5; *p < 0.05 vs. GS; °p < 0.05 vs. DM). (**b**) mRNA expression analysis of Wnt3, Wnt7b, Tbx4, Dll1 and Mesp2 in GS (black bars), DM (grey bars), DM +PTIO (gray striped bars). Data represented as mean fold increase ±s.e.m. compared to GS after subtraction of the housekeeping gene p0 signal (n=4; *p < 0.05 vs. GS; °p < 0.05 vs. DM). (**c**) ChIP/qRT-PCR of Zeb1 chromatin association on the promoter regions of Wnt3, Wnt7b, Tbx4, Dll1 and Mesp2. Chromatins extracted in GS (black bars), DM (grey bars) or NO (white bars). Data shown as mean fold increase ±s.e.m. compared to IgG value after Input normalization (n=3). (**d**) ChIP/qRT-PCR of Hdac1 chromatin association on the promoter regions of Wnt3, Wnt7b, Tbx4, Dll1 and Mesp2. Chromatins extracted in GS (black bars), DM (grey bars) or NO (white bars). Data shown as mean fold increase ±s.e.m. compared to IgG value after Input normalization (n=3). (**e**) ChIP/qRT-PCR of Hdac2 chromatin association on the promoter regions of Wnt3, Wnt7b, Tbx4, Dll1 and Mesp2. Chromatins extracted in GS (black bars), DM (grey bars) or NO (white bars). Data shown as mean fold increase ±s.e.m. compared to IgG value after Input normalization (n=3). (**f**) mRNA expression analysis of Wnt3, Wnt7b, Tbx4, Dll1 and Mesp2 in GS, DM and NO after nucleofection either with Scramble_LNA (GS: black bars, DM: grey bars and NO: white bars) or anti-miR-200b_LNA (DM: grey striped bars and NO: black striped bars). Data represented as mean fold increase ±s.e.m. compared to GS_Scramble LNA after subtraction of the housekeeping gene p0 signal (n=3; *p < 0.05 vs. GS_Scramble LNA; °p < 0.05 vs DM_Scramble LNA; § p < 0.05 anti-miR200b_LNA vs. Scramble LNA; # p < 0.05 vs. GS_Scramble LNA). Data analysed by Kolmogorov-Smirnov test.

Supplementary Fig. 5

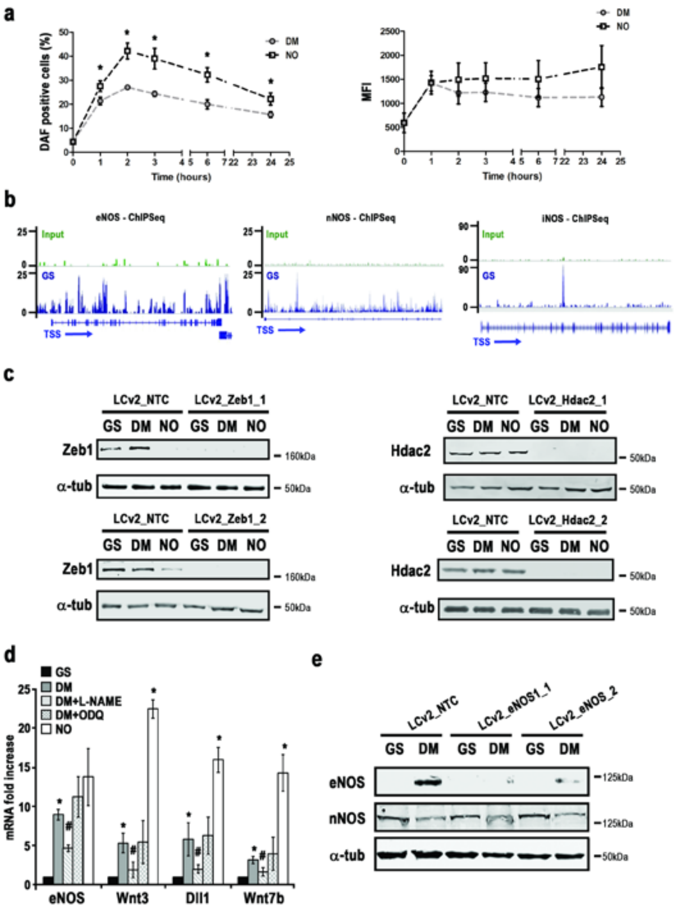

Supplementary Fig. 5 Zeb1 distribution on NOS promoters and mESC engineering by CRISPR/Cas9. (**a**) Left panel: percentage of DAF positive mESC cultured 1, 2, 3, 6, 24 h in DM or NO revealed by FACS analysis. Data represented as mean±s.e.m. (n=3; *p<0.05 vs DM). Right panel: DAF mean fluorescence intensity (MFI) measured by FACS analysis in mESC cultured 1, 2, 3, 6, 24 h in DM or NO. Data represented as mean±s.d. (n=3). (**b**) Sashimi plots showing Zeb1 (blue) peak distribution in the naïve mESC genome. Results represented according ChIP-seq data for eNOS (left blue panel), nNOS (middle blue panel) and iNOS (right blue panel) gene promoters. For each sashimi plot data are compared to input (dark green). (**c**) Left panel: representative western blot analysis showing Zeb1 inactivation in mESC extracts in GS, DM and NO prior (control vector LCv2_NTC) and after CRISPR/Cas9 inactivation of Zeb1 (LCv2_Zeb1_1 upper panels; LCv2_Zeb1_2 lower panels). Extracts probed with anti-Zeb1 antibody. α-tubulin antibody used as loading control for each extract (n=3). Full-length blot provided in Supplementary Fig. 14a. Right panel: representative western blot analysis showing Hdac2 inactivation in mESC extracts in GS, DM and NO prior (control vector LCv2_NTC) and after CRISPR/Cas9 inactivation of Hdac2 (LCv2_Hdac2_1 upper panels; LCv2_Hdac2_2 lower panels). Extracts probed with anti-Hdac2 antibody. α-tubulin used as loading control for each extract (n=3). Full-length blot provided in Supplementary Fig. 14b. (**d**) mRNA expression analysis of eNOS, Wnt3, Dll1 and Wnt7b in GS (black bars), DM (grey bars), DM + L-NAME (gray striped bars), DM + ODQ (gray squared bars) and NO (white bars). Data represented as mean fold increase ±s.e.m. compared to GS after subtraction of the housekeeping gene p0 signal (n=4; *p<0.05 vs. GS; #p<0.05 vs. DM). (**e**) Representative western blot analysis showing eNOS inactivation in mESC extracts in GS or DM prior (control vector LCv2_NTC) and after CRISPR/Cas9 inactivation of eNOS (LCv2_eNOS_1 and LCv2_eNOS_2). Extracts probed with eNOS and nNOS antibody. α-tubulin used as loading control for each extract (n=3). Full-length blot provided in Supplementary Fig. 14c. Data analysed by 2 way ANOVA (**a**) and Kolmogorov-Smirnov test (**d**).

Supplementary Fig. 6

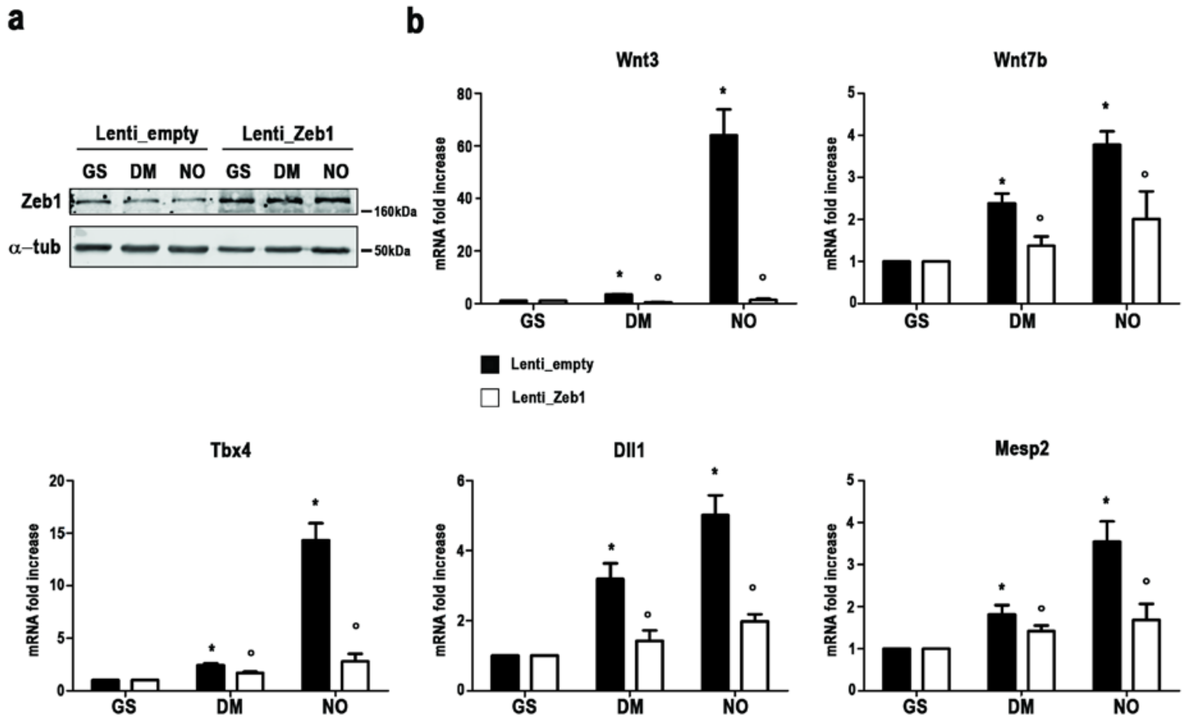

Supplementary Fig. 6 Zeb1 overexpression contrasts mesendoermal marker expression in released mESC. (**a**) Representative western blotting analysis of cell extracts obtained from mESC after infection with lentivirus expressing GFP (Lenti_Empty) or Zeb1 (Lenti_Zeb1) cultured 24 h in GS, DM and NO probed with Zeb1 antibody. For each extract, α-tubulin was used as loading control. The experiment was repeated three times. Full-length blot provided in Supplementary Fig. 15a. (**b**) mRNA expression analysis of Wnt3, Wnt7b, Tbx4, Dll1 and Mesp2 in mESC after infection with lentivirus expressing GFP (Lenti_Empty) or Zeb1 (Lenti_Zeb1) cultured 24 h in GS, DM and NO. Data are represented as mean fold increase ±s.e.m. compared to GS_Lenti_Empty after subtraction of the housekeeping gene p0 signal (n=3; *p < 0.05 vs. GS_Lenti_Empty; °p < 0.05 vs. Lenti_Empty). Data analysed by Kolmogorov-Smirnov test.

Supplementary Fig. 7

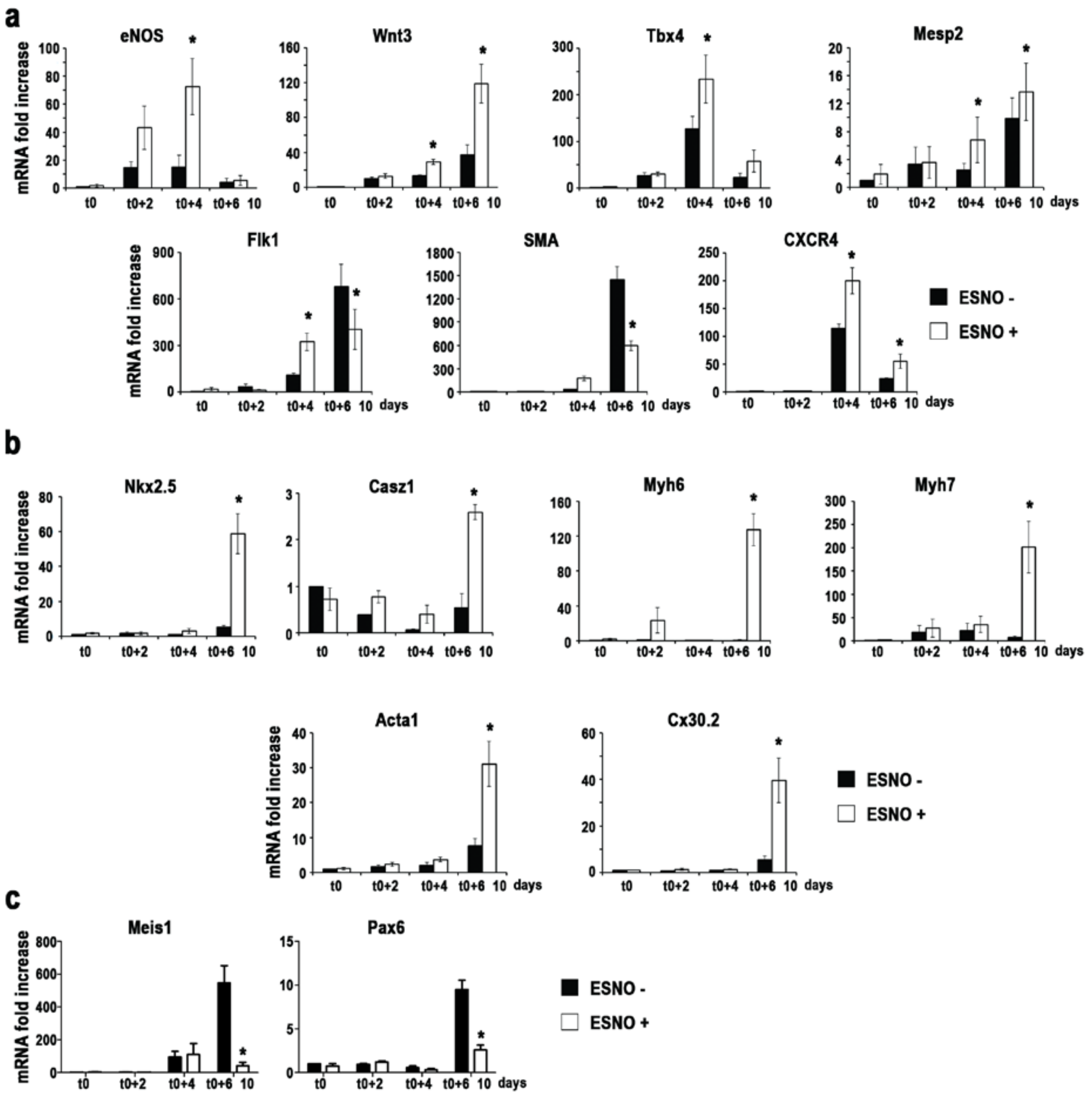

Supplementary Fig. 7 <u>Gene expression analysis of lineage associated markers in EBs obtained from ESNO+/- cells.</u> (**a**) qRT-PCR analysis of mesendoderm (Wnt3, Tbx4 and Mesp2) and vascular differentiation-associated markers (eNOS, Flk1, SMA and CXCR4) in total mRNA obtained from ESNO- (black bars) and ESNO+ (white bars) taken at different time points of the EB maturation process. Data are shown as mean ±s.e.m. fold increase compared to ESNO- derived EBs after subtraction of the housekeeping p0 gene signal (n=4 each time point; *p <0.05). (**b**) qRT-PCR analysis of cardiac associated markers (Nkx2.5, Casz1, Myh6, Acta1 and Cx30.2) in ESNO- (black bars) and ESNO+ (white bars) derived EBs taken at different time points of the EB maturation process. Data are shown as mean ±s.e.m. fold increase compared to ESNO- derived EBs after subtraction of the housekeeping p0 gene signal (n=4 each time point; *p <0.05). (**c**) qRT-PCR analysis of neuroectodermal associated markers (Meis1 and Pax6) in ESNO- (black bars) and ESNO+ (white bars) derived EBs taken at different time points of the EB maturation process. Data are shown as mean ±s.e.m. fold increase compared to ESNO- derived EBs after subtraction of the housekeeping p0 gene signal (n=4 each time point; *p <0.05). Data analysed by Kolmogorov-Smirnov test.

Supplementary Fig. 8

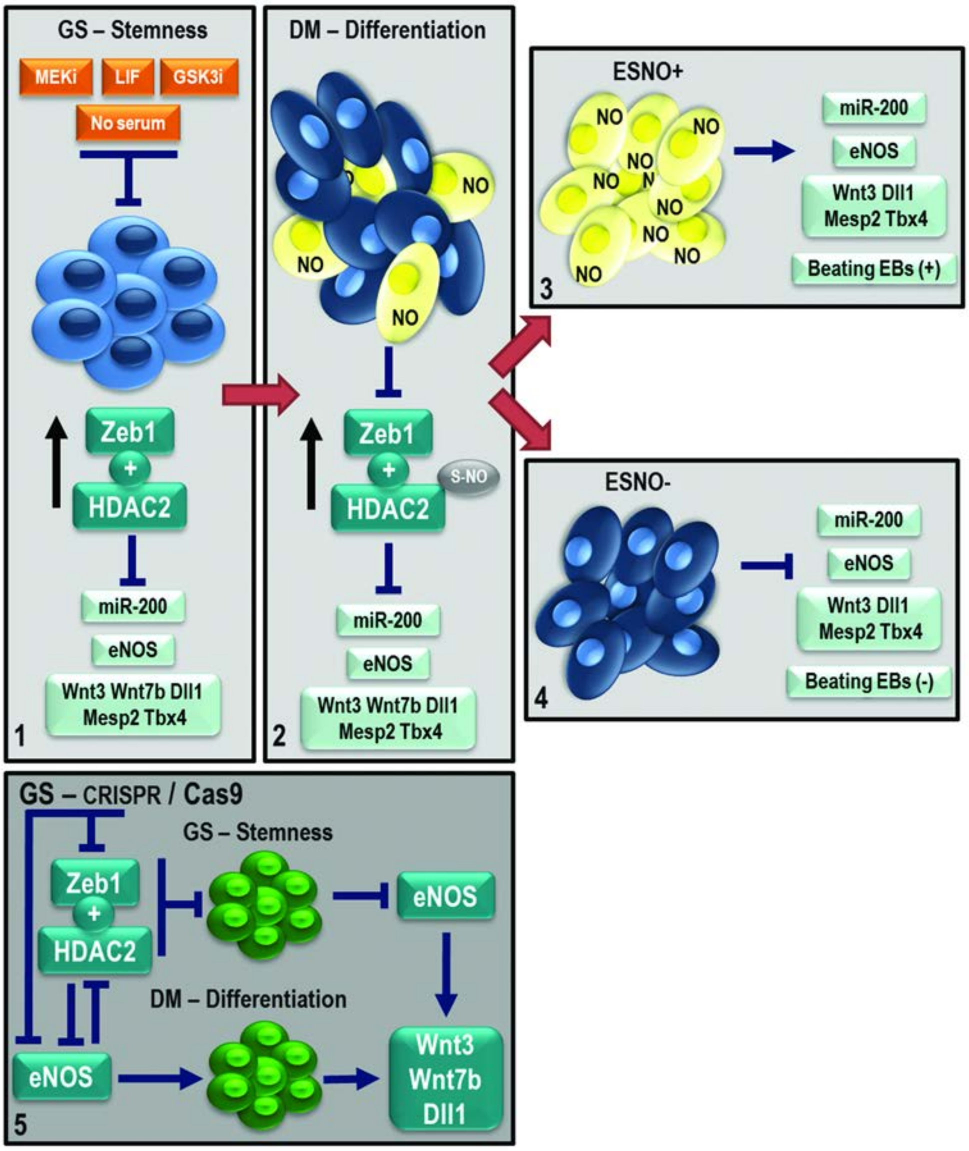

Supplementary Fig. 8 Schematic representation of the NO-dependent effects in naïve and differentiated mESC. In naïve mESC, which does not synthesize NO, the Zeb1:Hdac2 complex is bound to chromatin. This condition is associated with the ground state and the repression of lineage specific gene expression (panel 1). NO becomes detectable early after release from GS (DM). In these cells, Hdac2 become S-nitrosylated and the association of Zeb1 to chromatin is significantly reduced. Consistently, genes repressed by Zeb1, including miR-200 family members and transcripts belonging to the mesodermal lineage, are transcribed (panel 2). According to their higher (ESNO+) or lower (ESNO-) level of NO synthesis two mESC populations can be separated. ESNO+ cells express eNOS, mesendodermal/cardiovascular associated markers and form beating EBs (panel 3). ESNO- population remains negative for lineage specific markers and is unable to generate beating EBs (panel 4). Panel 5 shows that CRISPR/Cas9 engineering of naïve mESC by inactivating Zeb1 or Hdac2 induces eNOS as well as mesendodermal gene expression in undifferentiated cells. The inactivation of eNOS prevents the expression of mesendoderm-associated markers in mESC cultured in DM.

Supplementary Fig. 9 Related to Fig 3.

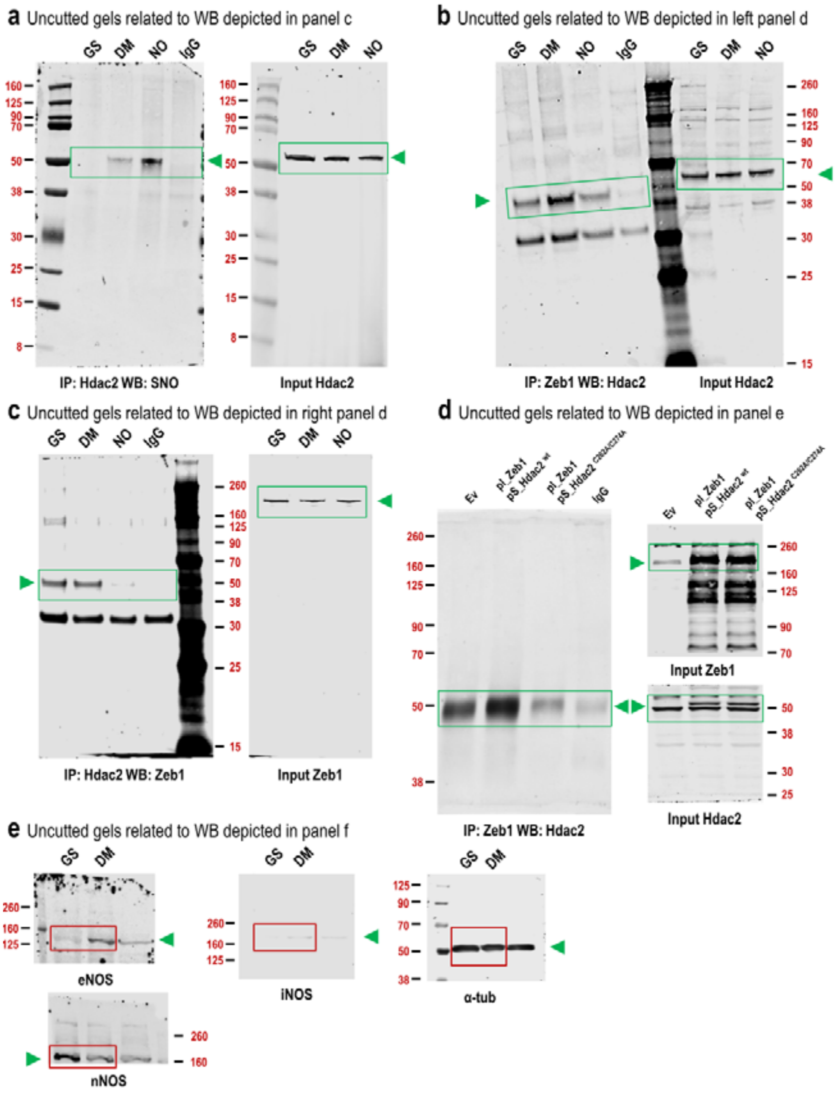

Supplementary Fig. 9 <u>Full-length images of representative Western blots related to Fig.3.</u> (**a**) Left panel: whole membrane blot of immunoprecipitation depicted in Fig. 3 panel c. Images of selected portions highlighted in the green frame. Right panel: whole membrane blot of immunoprecipitation input depicted in Fig. 3 panel c. Images of selected portions highlighted in the green frame. (**b**) Left panel: whole membrane blot of immunoprecipitation depicted in Fig. 3 left panel d. Images of selected portions highlighted in the green frame. Right panel: whole membrane blot of immunoprecipitation input depicted in Fig. 3 left panel d. Images of selected portions highlighted in the green frame. (**c**) Left panel: whole membrane blot of immunoprecipitation depicted in Fig. 3 right panel d. Images of selected portions highlighted in the green frame. Right panel: whole membrane blot of immunoprecipitation input depicted in Fig. 3 right panel d. Images of selected portions highlighted in the green frame. (**d**) Left panel: whole membrane blot of immunoprecipitation depicted in Fig. 3 panel e. Images of selected portions highlighted in the green frame. Right panel: whole membrane blot of immunoprecipitation input depicted in Fig. 3 panel e. Images of selected portions highlighted in the green frame. (**e**) Whole membrane blot of western blotting analysis related to eNOS, nNOS, iNOS and α-tubulin respectively depicted in Fig. 3 panel f. Images of selected portions highlighted in the red frame.

Supplementary Fig. 10 Related to Fig 5.

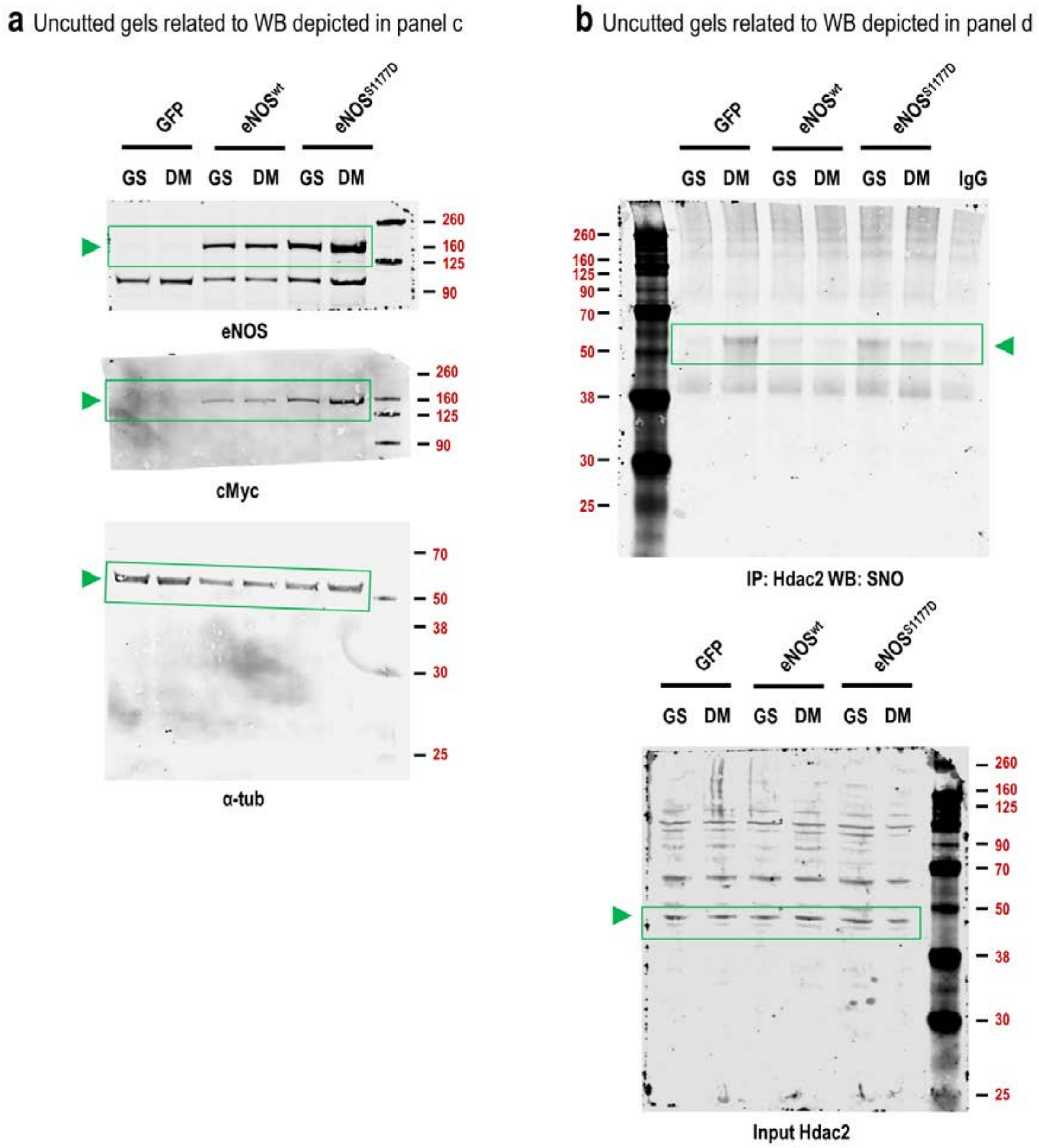

Supplementary Fig.10 Full-length images of representative Western blots related to Fig 5. **(a)** Whole membrane blot of western blotting analysis related to eNOS, cMyc and α-tubulin respectively depicted in Fig. 5 panel c. Images of selected portions highlighted in the green frame. **(b)** Upper panel: whole membrane blot of immunoprecipitation depicted in Fig. 5 panel d. Images of selected portions highlighted in the green frame. Lower panel: whole membrane blot of immunoprecipitation input depicted in Fig. 5 panel d. Images of selected portions highlighted in the green frame.

Supplementary Fig. 11 Related to Fig 6.

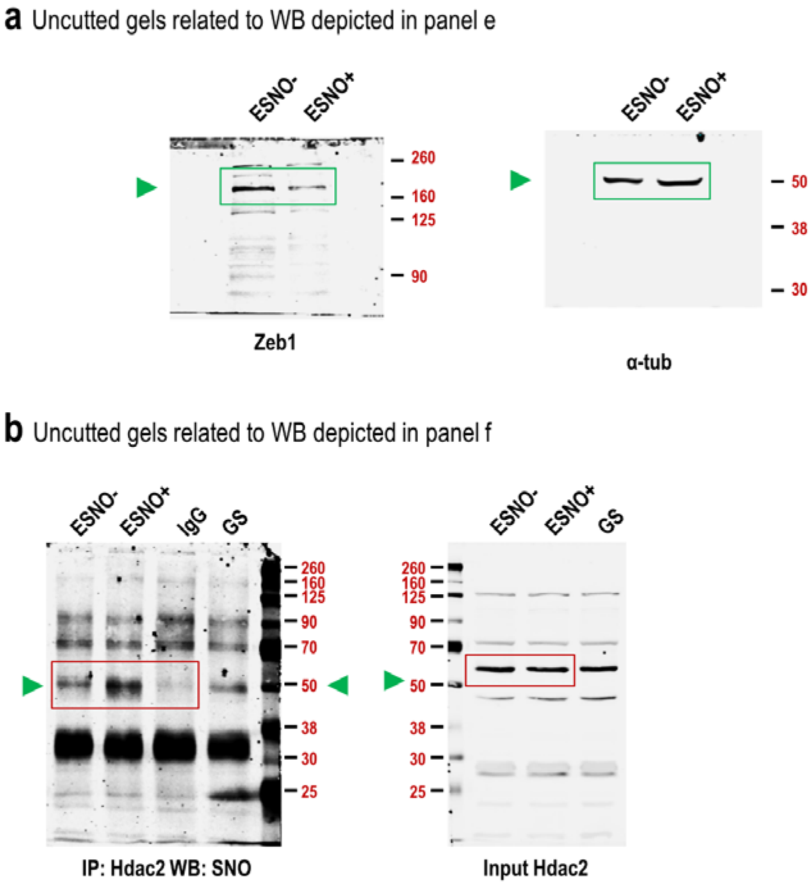

Supplementary Fig. 11 Full-length images of representative Western blots related to Fig 6. **(a)** Whole membrane blot of western blotting analysis related to Zeb1 and α-tubulin respectively depicted in Fig. 6 panel e. Images of selected portions highlighted in the green frame. **(b)** Left panel: whole membrane blot of immunoprecipitation depicted in Fig. 6 panel f. Images of selected portions highlighted in the red frame. Right panel: whole membrane blot of immunoprecipitation input depicted in Fig. 6 panel f. Images of selected portions highlighted in the red frame.

Supplementary Fig. 12 Related to Supplementary Fig 1.

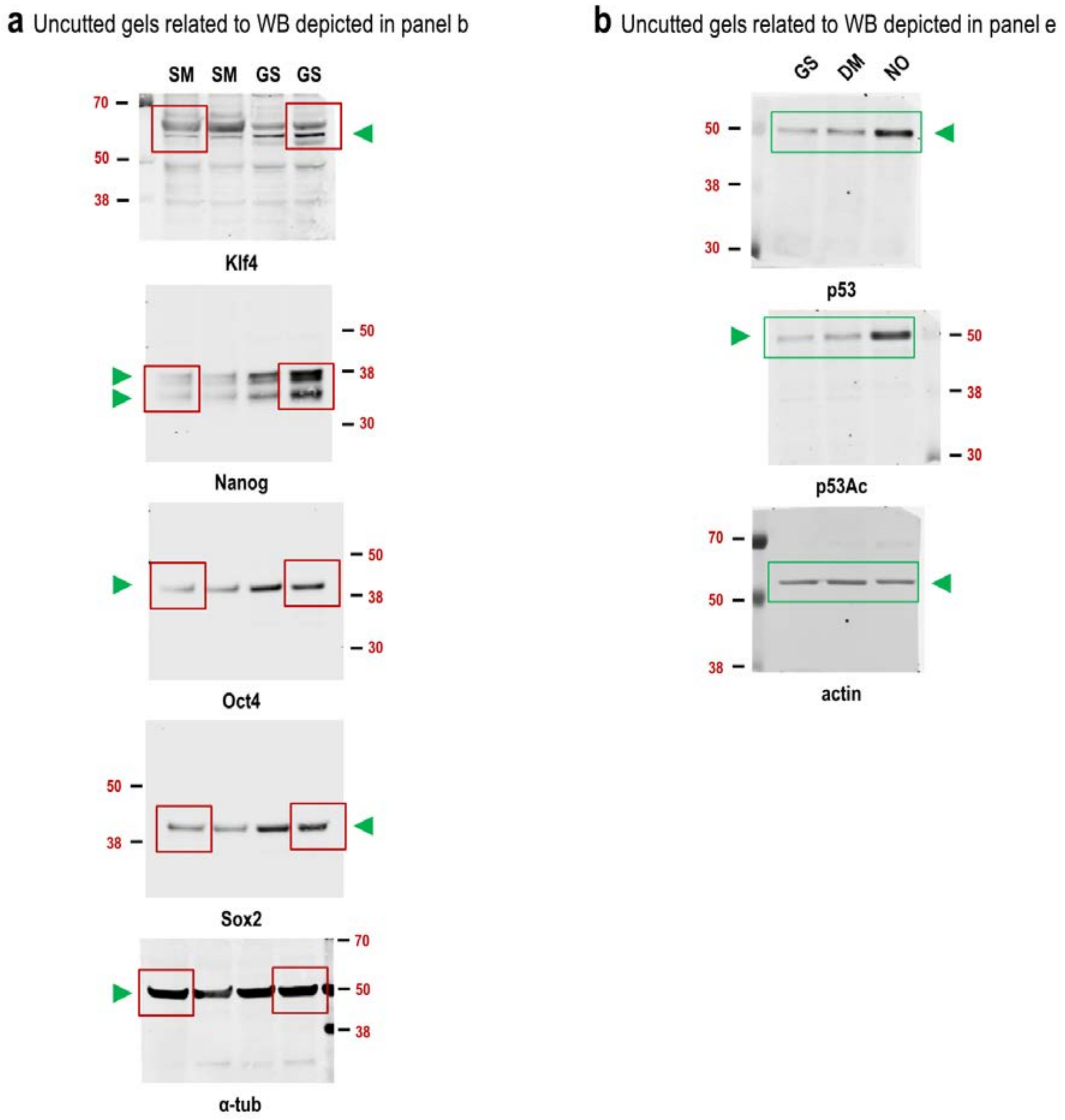

Supplementary Fig. 12 Full-length images of representative Western blots related to Supplementary Fig 1. **(a)** Whole membrane blot of western blotting analysis related to Klf4, Nanog, Oct4, Sox2 and α-tubulin respectively depicted in Suppl. Fig. 1 panel b. Images of selected portions highlighted in the red frame. **(b)** Whole membrane blot of western blotting analysis related to p53, p53Ac and actin respectively depicted in Suppl. Fig. 1 panel e. Images of selected portions highlighted in the green frame.

Supplementary Fig. 13 Related to Supplementary Fig 3.

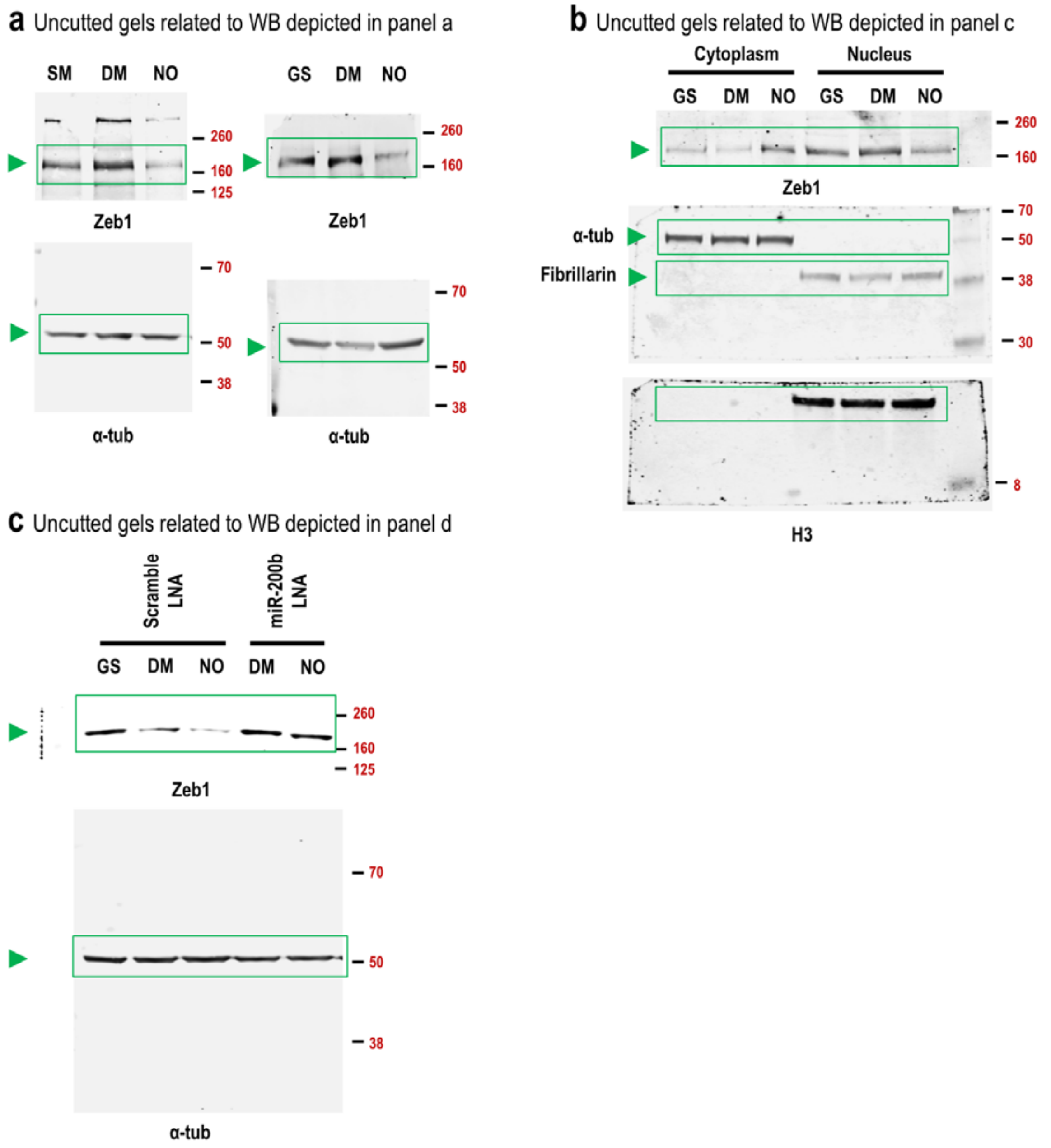

Supplementary Fig. 13 Full-length images of representative Western blots related to Supplementary Fig 3. **(a)** Whole membrane blot of western blotting analysis related to Zeb1 and α-tubulin respectively depicted in Suppl. Fig. 2 panel a. Images of selected portions highlighted in the green frame. **(b)** Whole membrane blot of western blotting analysis related to Zeb1 and α-tubulin respectively depicted in Suppl. Fig. 2 panel b. Images of selected portions highlighted in the green frame. **(c)** Whole membrane blot of western blotting analysis related to Zeb1, α-tubulin, Fibrillarin and H3 respectively depicted in Suppl. Fig. 2 panel c. Images of selected portions highlighted in the green frame.

Supplementary Fig. 14 Related to Supplementary Fig 5.

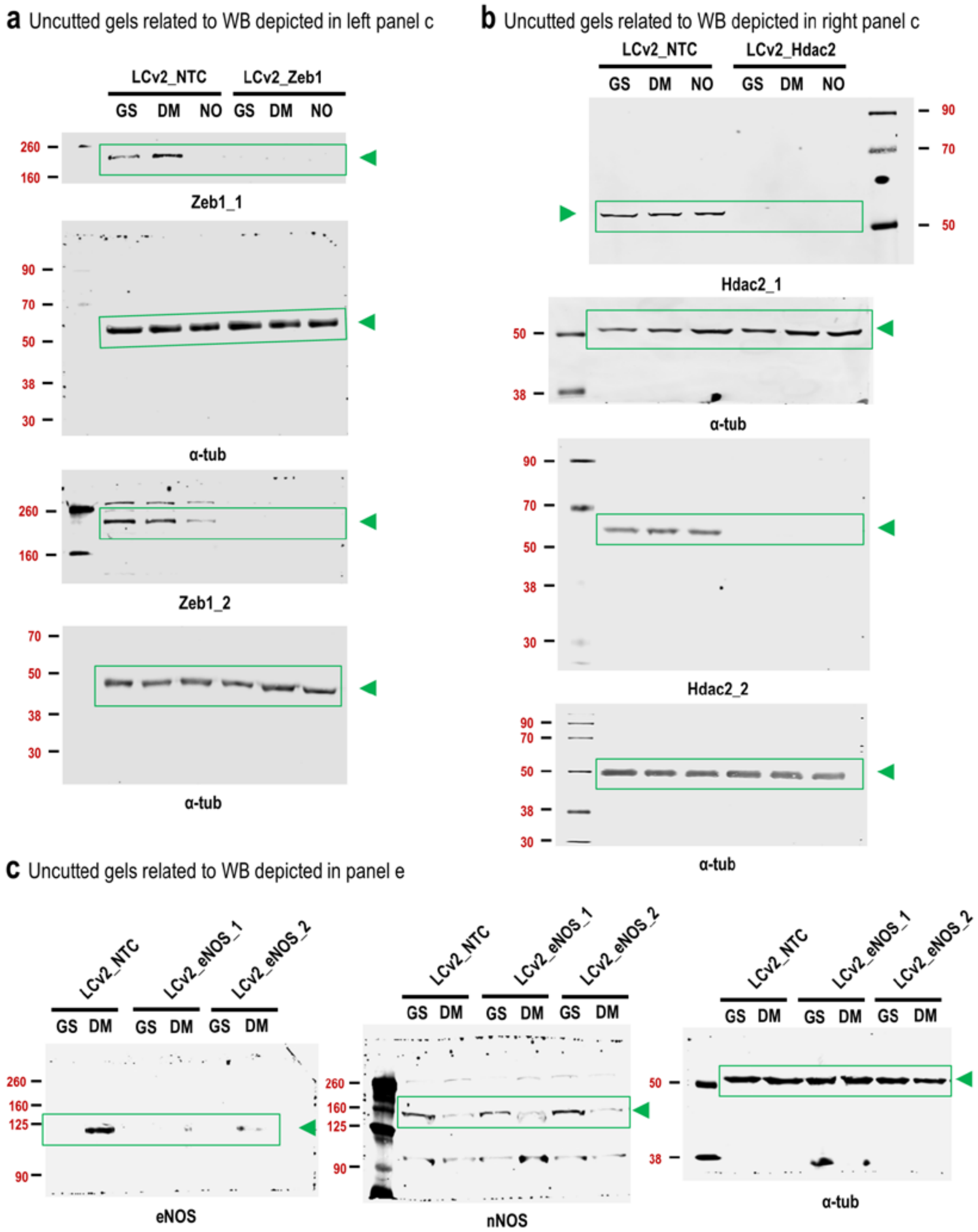

Supplementary Fig. 14 Full-length images of representative Western blots related to Supplementary Fig 5. **(a)** Whole membrane blot of western blotting analysis related to Zeb1 and α-tubulin respectively depicted in Suppl. Fig. 5 left panel c. Images of selected portions highlighted in the green frame. Upper panels related to LCv2_Zeb1_1 and lower panels related to LCv2_Zeb1_2 both compared to LCv2_NTC. **(b)** Whole membrane blot of western blotting analysis related to Hdac2 and α-tubulin respectively depicted in Suppl. Fig. 5 right panel c. Images of selected portions highlighted in the green frame. Upper panels related to LCv2_Hdac2_1 and lower panels related to LCv2_Hdac2_2 both compared to LCv2_NTC. **(c)** Whole membrane blot of western blotting analysis related to eNOS, nNOS and α-tubulin respectively depicted in Suppl. Fig. 5 panel e. Images of selected portions highlighted in the green frame.

Supplementary Fig. 15 Related to Supplementary Fig 6.

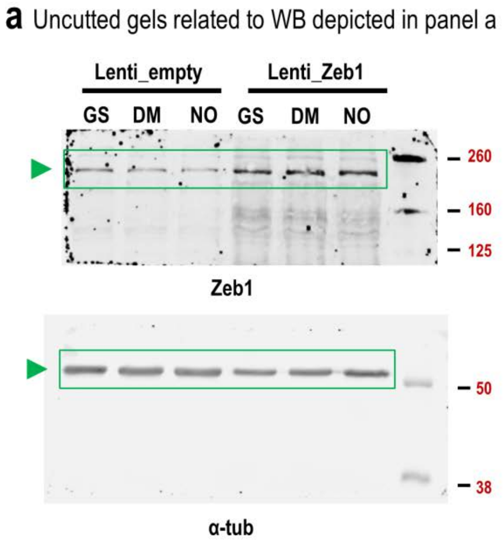

Supplementary Fig. 15 Full-length images of representative Western blots related to Supplementary Fig 6. **(a)** Whole membrane blot of western blotting analysis related to Zeb1 and α-tubulin respectively depicted in Suppl. Fig. 6 panel a. Images of selected portions highlighted in the green frame.

Supplementary tables

Supplementary Table 1. General sequencing statistics

Sample	Experiment	Raw Reads [#]	Trimmed Reads [#]	Mean Length Trimmed Reads [nt]	Mapped Reads [#]
GS_1	RNA Seq	45773515	41440710	111	32764129
GS_2	RNA Seq	60336017	54614154	106	39649345
GS_3	RNA Seq	37234577	34938551	115	27307705
DM^{GS}_1	RNA Seq	51692848	46676326	110	38380896
DM^{GS} _2	RNA Seq	63964694	58385924	101	47875495
DM^{GS} _3	RNA Seq	50253980	42234799	109	34039478
DM^{GSNO}_1	RNA Seq	59883454	55809069	119	45833565
DM^{GSNO} _2	RNA Seq	40472427	36594367	107	26592224
DM^{GSNO} _3	RNA Seq	41609238	34676920	109	25606368
GS_ctrl_1 Input	ChIP Seq	50657015	49386645	116	23162440
GS_Zeb1_1 Zeb1_IP	ChIP Seq	38415268	37519506	113	19823308
GS_Zeb1_2 Zeb1_IP	ChIP Seq	30630621	30043393	115	20762694
GS_Zeb1_3 Zeb1_IP	ChIP Seq	30909628	30244202	114	19787120

Supplementary Table 2. List of selected genes regulated in each group depicted in Fig. 1A.

Ensembl Gene Id	Gene Name	UniProt Protein Description	Chr	Gene Start	Gene End	Str
ENSMUSG00000038242	Aox4	Aldehyde oxidase 4	1	58210397	58268597	+
ENSMUSG00000026173	Plcd4	1-phosphatidylinositol 4,5-bisphosphate phosphodiesterase delta-4	1	74542888	74567794	+
ENSMUSG00000033227	Wnt6	Protein Wnt-6	1	74771892	74785322	+
ENSMUSG00000026249	Serpine2	Glia-derived nexin	1	79794197	79861180	-
ENSMUSG00000084989	E030010 N08Rik	Protein E030010N08Rik	1	93168725	93231072	+
ENSMUSG00000047793	Sned1	Sushi, nidogen and EGF-like domain-containing protein 1	1	93235841	93301065	+
ENSMUSG00000042268	Slc26a9	Solute carrier family 26 member 9	1	131744022	131771504	+
ENSMUSG00000097993	Ptprv		1	135108497	135132594	-
ENSMUSG00000006411	Pvrl4	Poliovirus receptor-related protein 4	1	171370099	171388598	+
ENSMUSG00000075304	Sp5	Transcription factor Sp5	2	70474923	70477729	+
ENSMUSG00000074968	Ano3	Anoctamin	2	110655201	110950923	-
ENSMUSG00000040093	Bmf	Bcl-2-modifying factor	2	118528757	118549687	-
ENSMUSG00000027400	Pdyn	Proenkephalin-B	2	129686565	129699844	-
ENSMUSG00000027401	Tgm3	Protein-glutamine gamma-glutamyltransferase E	2	130012349	130050399	+
ENSMUSG00000027356	Fermt1	Fermitin family homolog 1	2	132904389	132945906	-
ENSMUSG00000053141	Ptprt	Receptor-type tyrosine-protein phosphatase T	2	161521990	162661147	-
ENSMUSG00000017817	Jph2	Junctophilin-2	2	163336242	163397993	-
ENSMUSG00000017697	Ada	Adenosine deaminase	2	163726584	163750239	-
ENSMUSG00000028640	Tfap2c	Transcription factor AP-2 gamma	2	172549593	172558622	+
ENSMUSG00000027570	Col9a3	Procollagen	2	180597790	180622189	+
ENSMUSG00000027580	Helz2	Helicase with zinc finger domain 2	2	181227615	181242027	-
ENSMUSG00000069114	Zbtb10	Protein Zbtb10	3	9250602	9285333	+
ENSMUSG00000045092	S1pr1	Sphingosine 1-phosphate receptor 1	3	115710433	115715072	-
ENSMUSG00000040998	Npnt	Nephronectin	3	132881745	132950291	-
ENSMUSG00000028435	Aqp3	Aquaporin-3	4	41092722	41098183	-
ENSMUSG00000028487	Bnc2	Zinc finger protein basonuclin-2	4	84275095	84675275	-

ENSMUSG00000006386	Tek	Angiopoietin-1 receptor Endothelial-specific receptor tyrosine kinase	4	94739289	94874976	+
ENSMUSG00000028789	Adc	Antizyme inhibitor 2	4	128930233	128962442	-
ENSMUSG00000040533	Matn1	Cartilage matrix protein	4	130944385	130955475	+
ENSMUSG00000028860	Sytl1	Synaptotagmin-like protein 1	4	133253090	133263113	-
ENSMUSG00000029055	Plch2	1-phosphatidylinositol 4,5-bisphosphate phosphodiesterase eta-2	4	154983115	155056784	-
ENSMUSG00000034981	Parm1	Prostate androgen-regulated mucin-like protein 1 homolog	5	91517615	91626894	+
ENSMUSG00000034842	Art3	Ecto-ADP-ribosyltransferase 3	5	92331827	92414628	+
ENSMUSG00000042078	Svop	Synaptic vesicle 2-related protein	5	114026910	114091570	-
ENSMUSG00000024211	Grm8	Metabotropic glutamate receptor 8	6	27275119	28135178	-
ENSMUSG00000003476	Crhr2	Corticotropin-releasing factor receptor 2	6	55090049	55133016	-
ENSMUSG00000030064	Frmd4b	FERM domain-containing protein 4B	6	97286867	97617541	-
ENSMUSG00000035357	Pdzrn3	E3 ubiquitin-protein ligase PDZRN3	6	101149609	101377897	-
ENSMUSG00000030111	A2m	Alpha-2-macroglobulin-P	6	121635376	121679227	+
ENSMUSG00000078776	9530053 A07Rik	Protein 9530053A07Rik	7	28129466	28164811	+
ENSMUSG00000036915	Kirrel2	Kin of IRRE-like protein 2	7	30447534	30457690	-
ENSMUSG00000009487	Otog	Otogelin	7	46240987	46311434	+
ENSMUSG00000030523	Trpm1	Transient receptor potential cation channel subfamily M member 1	7	64153835	64269775	+
ENSMUSG00000035314	Gdpd5	Glycerophosphodiester phosphodiesterase domain-containing protein 5	7	99381549	99460983	+
ENSMUSG00000005686	Ampd3	AMP deaminase 3	7	110768206	110812405	+
ENSMUSG00000030669	Calca	Calcitonin	7	114631478	114636357	-
ENSMUSG00000051457	Spn	Leukosialin	7	127132232	127137823	-
ENSMUSG00000048528	Nkx1-2	NK1 transcription factor-related protein 2	7	132594878	132599637	-
ENSMUSG00000041309	Nkx6-2	NK6 transcription factor related	7	139579376	139582797	-
ENSMUSG00000066129	Kndc1	Protein very KIND	7	139894696	139941537	+
ENSMUSG00000025473	Adam8	Disintegrin and metalloproteinase domain-containing protein 8	7	139978932	139992562	-
ENSMUSG00000031502	Col4a1	Collagen alpha-1(IV) chain	8	11198423	11312826	-

ENSMUSG00000031549	Ido2	Indoleamine 2,3-dioxygenase 2	8	24531894	24576333	-
ENSMUSG00000031530	Dusp4	Dual specificity protein phosphatase 4	8	34807297	34819894	+
ENSMUSG00000045333	Zfp423	Zinc finger protein 423 Zinc finger protein 423	8	87661810	87959595	-
ENSMUSG00000074151	Nlrc5	Protein NLRC5	8	94472763	94527272	+
ENSMUSG00000031886	Ces2e	Pyrethroid hydrolase Ces2e	8	104926260	104934672	+
ENSMUSG00000043251	Exoc3l	Exocyst complex component 3-like protein	8	105289924	105296098	-
ENSMUSG00000032118	Fez1	Fasciculation and elongation protein zeta-1	9	36821939	36878924	+
ENSMUSG00000032125	Robo4	Roundabout homolog 4	9	37401897	37414023	+
ENSMUSG00000049281	Scn3b	Sodium channel subunit beta-3	9	40269216	40291618	+
ENSMUSG00000032135	Mcam	Cell surface glycoprotein MUC18	9	44134469	44142727	+
ENSMUSG00000032087	Dscaml1	Down syndrome cell adhesion molecule-like protein 1 homolog	9	45430293	45753164	+
ENSMUSG00000032060	Cryab	Alpha-crystallin B chain	9	50752758	50756633	+
ENSMUSG00000032232	Cgnl1	Cingulin-like 1 Cingulin-like protein 1	9	71626509	71771602	-
ENSMUSG00000086236	5830418 P13Rik		9	103423578	103461260	+
ENSMUSG00000032591	Mst1	Hepatocyte growth factor-like protein	9	108080436	108085003	+
ENSMUSG00000039115	Itga9	Protein Itga9	9	118606690	118901003	+
ENSMUSG00000019817	Plagl1	Protein Plagl1	10	13060504	13131694	+
ENSMUSG00000019851	Perp	p53 apoptosis effector related to PMP-22	10	18845071	18857073	+
ENSMUSG00000037440	Vnn1	Pantetheinase	10	23894688	23905343	+
ENSMUSG00000019989	Enpp3	Ectonucleotide pyrophosphatase/phosphodiesterase family member 3	10	24773814	24836195	-
ENSMUSG00000038916	Soga3	Protein SOGA3	10	29143996	29199630	+
ENSMUSG00000000731	Aire	Autoimmune regulator	10	78030022	78043610	-
ENSMUSG00000005355	Casp14	Caspase-14	10	78711997	78718293	-
ENSMUSG00000004359	Spic	Transcription factor Spi-C	10	88674772	88685015	-
ENSMUSG00000058589	Anks1b	Ankyrin repeat and sterile alpha motif domain-containing protein 1B	10	89873509	90973300	+
ENSMUSG00000019960	Dusp6	Dual specificity protein phosphatase 6	10	99263231	99267488	+
ENSMUSG00000025407	Gli1	Zinc finger protein GLI1	10	127329889	127341589	-

ENSMUSG00000020122	Egfr	Epidermal growth factor receptor	11	16752203	16918158	+
ENSMUSG00000018339	Gpx3	Glutathione peroxidase	11	54902453	54910377	+
ENSMUSG00000005951	Shpk	Sedoheptulokinase	11	73199460	73224511	+
ENSMUSG00000017390	Aldoc	Fructose-bisphosphate aldolase C	11	78322968	78327781	+
ENSMUSG00000018845	Unc45b	Protein unc-45 homolog B	11	82910550	82943403	+
ENSMUSG00000001555	Fkbp10	Peptidyl-prolyl cis-trans isomerase FKBP10	11	100415697	100424824	+
ENSMUSG00000019326	Aoc3	Membrane primary amine oxidase	11	101330605	101341938	+
ENSMUSG00000025161	Slc16a3	Monocarboxylate transporter 4	11	120948480	120960868	+
ENSMUSG00000020598	Nrcam	Neuronal cell adhesion molecule	12	44328885	44601846	+
ENSMUSG00000035105	Egln3	Egl nine homolog 3	12	54178981	54203860	-
ENSMUSG00000021298	Gpr132	Probable G-protein coupled receptor 132	12	112850876	112860916	-
ENSMUSG00000004341	Gpx6	Glutathione peroxidase 6	13	21312217	21319624	+
ENSMUSG00000051335	Gfod1	Glucose-fructose oxidoreductase domain-containing protein 1	13	43195519	43304172	-
ENSMUSG00000042102	Dmgdh	Dimethylglycine dehydrogenase	13	93674433	93752831	+
ENSMUSG00000021662	Arhgef28	Rho guanine nucleotide exchange factor 28 Rho-guanine nucleotide exchange factor	13	97898595	98206165	-
ENSMUSG00000021950	Anxa8	Annexin A8	14	34085981	34100571	+
ENSMUSG00000021848	Otx2	Homeobox protein OTX2	14	48657679	48667644	-
ENSMUSG00000000958	Slc7a7	Y+L amino acid transporter 1	14	54369442	54417780	-
ENSMUSG00000022091	Sorbs3	Vinexin	14	70180468	70207637	-
ENSMUSG00000033644	Piwil2	Piwi-like protein 2	14	70372480	70429094	-
ENSMUSG00000022096	Hr	Protein hairless	14	70552212	70573548	+
ENSMUSG00000005360	Slc1a3	Excitatory amino acid transporter 1	15	8634124	8710764	-
ENSMUSG00000022197	Pdzd2	Protein Pdzd2	15	12359711	12739924	-
ENSMUSG00000022286	Grhl2	Grainyhead-like protein 2 homolog	15	37233036	37363569	+
ENSMUSG00000058099	Nfam1	NFAT activation molecule 1	15	82997721	83033306	-
ENSMUSG00000062760	1810041 L15Rik	Protein 1810041L15Rik	15	84379203	84447097	-
ENSMUSG00000037353	Letmd1	LETM1 domain-containing protein 1	15	100469034	100479252	+
ENSMUSG00000043391	2510009 E07Rik	UPF0524 protein C3orf70 homolog	16	21649045	21694665	-

ENSMUSG00000022912	Pros1	Vitamin K-dependent protein S	16	62854307	62929346	+
ENSMUSG00000041134	Cyyr1	Cysteine and tyrosine-rich protein 1	16	85421533	85553397	-
ENSMUSG00000005251	Ripk4	Receptor-interacting serine/threonine-protein kinase 4	16	97741933	97763737	-
ENSMUSG00000023827	Agpat4	1-acyl-sn-glycerol-3-phosphate acyltransferase delta	17	12118704	12219645	+
ENSMUSG00000037321	Tap1	Antigen peptide transporter 1	17	34187553	34197225	+
ENSMUSG00000073418	C4b	Complement C4-B	17	34728380	34743882	-
ENSMUSG00000023913	Pla2g7	Platelet-activating factor acetylhydrolase	17	43568098	43612201	+
ENSMUSG00000039193	Nlrc4	NLR family CARD domain-containing protein 4	17	74426295	74459108	-
ENSMUSG00000024427	Spry4	Protein sprouty homolog 4	18	38586268	38601268	-
ENSMUSG00000024619	Cdx1	Homeobox protein CDX-1	18	61018862	61036199	-
ENSMUSG00000046318	Ccbe1	Collagen and calcium-binding EGF domain-containing protein 1	18	66045302	66302739	-
ENSMUSG00000036098	Myrf	Myelin regulatory factor	19	10208272	10240748	-
ENSMUSG00000031160	Eras	GTPase ERas	X	7924276	7928607	-
ENSMUSG00000002006	Pdzd4	PDZ domain-containing protein 4	X	73793359	73824969	-
ENSMUSG00000046774	8030474 K03Rik	Protein 8030474K03Rik	X	101794656	101798642	+
ENSMUSG00000009596	Taf7l	Transcription initiation factor TFIID subunit 7-like	X	134460118	134476490	-

(basemean >5, fdr < 0.05, log2fc > ± 2x)

Supplementary Table 3. EB diameter measures.

	t0 + 2 days		t0 + 6 days	
	**DAF - EBs**	**DAF + EBs**	**DAF - EBs**	**DAF + EBs**
**Average**	223.46 µm	350.61 µm	527.54 µm	793.02 µm
**SE**	70.54	24.54	138.95	168.40
**N**	20	30	12	12
**p**	0.05 (*)		0.11	

Supplementary Table 4. Antibody list

**Antibody**	**Company**	**Cat. number**	**Host**	**Clonality**	**Application**
eNOS	Becton Dickinson	610296	Ms	mAb	WB
Fibrillarin	Santa Cruz	SC374022	Ms	mAb	WB
Histone 3 (H3)	Cell Signaling	9715	Rb	pAb	WB
Hdac1 (10E2)	Abcam	ab46985	Ms	mAb	ChIP
Hdac2 (H54)	Santa Cruz	SC7899	Rb	pAb	WB, IP
Hdac2	GeneTex	GTX109642	Rb	pAb	ChIP
iNOS	Cell Signaling	2982	Rb	pAb	WB
Klf4	Abcam	ab72543	Rb	pAb	WB
Myc-Tag (9B11)	Cell Signaling	2276	Ms	mAb	WB
Nanog	Abcam	ab80892	Rb	pAb	WB
nNOS	Cell Signaling	4234	Rb	pAb	WB
Oct4	Abcam	ab19857	Rb	pAb	WB
p53 (Pab240)	Santa Cruz	SC99	Ms	mAb	WB, ChIP
Acetyl-p53 (Lys379)	Cell Signaling	2570	Rb	pAb	WB
S-Nitroso-Cysteine (SNO-Cys)	Alpha Diagnostic	NISC11-A	Rb	pAb	IP, WB
Sox2	Abcam	ab97959	Rb	pAb	WB
Zeb1	Active motif	61119	Rb	pAb	IP
Zeb1 (H-102)	Santa Cruz	SC25388	Rb	mAb	WB, ChIP
α-tubulin (DM1A)	Cell Signaling	3873	Ms	mAb	WB
β-Actin (6-11B-1)	SIGMA-ALDRICH	T6793	Ms	mAb	WB

Ms=mouse; Rb=rabbit; mAb=monoclonal antibody; pAb=polyclonal antibody; WB=western blotting; IP=immunoprecipitation; ChIP=chromatin immunoprecipitation.

Supplementary table 5. qRT-PCR primer list

Gene	Forward primer	Reverse primer
Wnt3	5’-CAGATGCCCGCTCAGCTATGAACA-3’	5’-AGCAGCACCAGTGGAAGACGCAAT-3’
Wnt7b	5’-GTCAGGGATGTTTGTCCCACTTG-3’	5’-TCTGGTAGGTCCTTGTGCCACTC-3’
Dll1	5’-GATACACACAGCAAACGTGACACC-3’	5’-TTCCATCTTACACCTCAGTCGCTA-3’
Tbx4	5′-GCTTCACTTATATGG-TACTCAG-3′	5′-ACGGTCAATGGGGGAAGAAGG-3′
Mesp2	5′-CCGGTCCAGCTTCCCAGAGTCACACC-3′	5′-GGACACCCCACTACTCATGGCTGA-3′
eNOS	5′-CATGAGTTCAGAGATTGGCATGAG-3′	5′-GGACACCCCACTACTCATGGCTGA-3′
nNOS	5′-CCGCCAAAACCTGCAAAG-3′	5′-TGTGGAGACCACGAAGATG-3′
iNOS	5′-CAACTACTGCTGGTGGTGACA-3′	5′-TGAAGGTGTGGTTGAGTTCTCT-3′
Flk1	5´-CCAGTGGTACTGGCAGCTAGAA-3´	5´-TTTCCCCCCTGGAAATCCT-3´
SMA	5´-CCAGCACCATGAAGATCAAG-3´	5´-TGGAAGGTAGACAGCGAAGC-3´
CXCR4	5´-CCGGGATGAAAACGTCCAT-3´	5´-TTGCCGACTATGCCAGTCAA-3`
Nkx2.5	5´-GCTACAAGTGCAAGCGACAG-3´	5´-GGGTAGGCGTTGTAGCCATA-3´
Myh6	5´-CTCATGCGCATTGAGTTCAAG-3´	5´-CGAATGTTCCACTGGATAACCA-3´
Myh7	5´-TCTGCTGAAGGACACTCAAATCC-3´	5´-GTTCTCTTTCAGGTCGTCATTGG-3´
Acta1	5´-GCCAGAGTCAGAGCAGCAGA-3´	5´-TTGCTCTGGGCCTCATCACC-3´
Cx30.2	5´-CACTACCGCTTCTGGCTCTT-3´	5´-TGGAGTAGATGACGAACAGCA-3`
Casz1	5´-CTGCCTCCATCATTGAGAGAAT-3´	5´-TCATTACTGGGATCCTTCACG-3´
Olig2	5´-GTGGCTTCAAGTCATCTTCC-3`	5`-GTAGATCTCGCTCACCAGTC-3`
Meis1	5´-CGAGCAGTCAGCCAAGGGAC-3`	5`-TCAGTCACCATTGTAGACAACG-3`
Pax6	5`-AACACCAACTCCATCAGTTC-3`	5´-ATCTGGATAATGGGTCCTCT-3`
p0	5′-GCGTCCTGGCATTGTCTGT-3′	5′-GAAGGCCTTGACCTTTTCAGTAAG-3′

Supplementary table 6. ChIP-qRT-PCR primer list

Gene	Forward primer	Reverse primer
miR200 cluster 1	5´-GGTGGGCCCCTAGGAGTCT-3´	5´-ATCTGCAGCCCCAAGTCTGT-3´
Dll1	5´-GGTCCTCGGTGTGTGTTCTC-3´	5´-CGCTCCCAGAGTGTTCTA-3´
Mesp2	5´-AGTAGCAGGGCGGAGTGAAG-3´	5´-GGTGTCAAAACCCACAGAGC-3´
Tbx4	5´-CTGGGGGACTCTTCACAG-3´	5´-CATCTCCTGCGGGCACAG-3´
Wnt7b	5´-TCCACATTCCTGAACCCAG-3´	5´-CGTTCGGTCACACTGCC-3´
Wnt3	5´-GATTGGCTGCTCGCTGACATCC-3´	5´-CTTGAGGTTGGAAATGACTTTC-3´
eNOS	5´-TAGGGCTGTGCGGCAAGCA-3´	5´-TGGTCGGGTTGGGGACGG-3´

Supplementary table 7. Target-specific sgRNAs for CRISPR/Cas9 technology

Gene	Forward primer	Reverse primer
Zeb1_1	5´-caccgCCCGAGGAAGACCAGCGGC-3´	5´-aaac-GCCGCTGGTCTTCCTCGGGc-3´
Zeb1_2	5´-caccgCTTGATGCCTGTGAATGGC-3´	5´-aaacGCCATTCACAGGCATCAAGc-3´
Hdac2_1	5´-caccgAACGTCGGAGAAGATTGTC-3´	5´-aaacGACAATCTTCTCCGACGTTc-3´
Hdac2_2	5´-caccgATATGGCTGTCAATTGGGC-3´	5´-aaacGCCCAATTGACAGCCATATc-3´
eNOS_1	5´-caccgAGACGCTGCTTGGGATCCC-3´	5´-aaacGGGATCCCAAGCAGCGTCTc-3´
eNOS_2	5´-caccgGTTTGGGGCCAAGCAGGCC-3´	5´-aaacGGCCTGCTTGGCCCCAAACc-3´
NTC	5'-caccgTTCCGGGCTAACAAGTCCT-3'	5'-aaacAGGACTTGTTAGCCCGGAAc-3'
